# Editorial: Casein kinases in human diseases

**DOI:** 10.3389/fmolb.2022.1094922

**Published:** 2022-12-02

**Authors:** Andrea Venerando, Victor H. Bustos, Lorenzo A. Pinna, Giorgio Cozza

**Affiliations:** ^1^ Department of Comparative Biomedicine and Food Science, Agripolis Campus, University of Padova, Legnaro, Italy; ^2^ Fisher Drug Discovery Resource Center, The Rockefeller University, New York, NY, United States; ^3^ CNR Neuroscience Institute, Padova, Italy; ^4^ Department of Molecular Medicine, University of Padova, Padova, Italy

**Keywords:** casein kinase, CK1, CK2, Fam20C, protein kinase, phosphorylation, G-CK

## Introduction

140 years after the discovery of the first phosphoprotein (i.e., casein), the research on protein kinases (in humans about 500 enzymes that catalyse the transfer of phosphate from ATP to proteins) represents an evergreen field in the scientific landscape and the journey into the dissection of their crucial biological roles continues to unveil new breath-taking scenarios.

**FIGURE 1:**
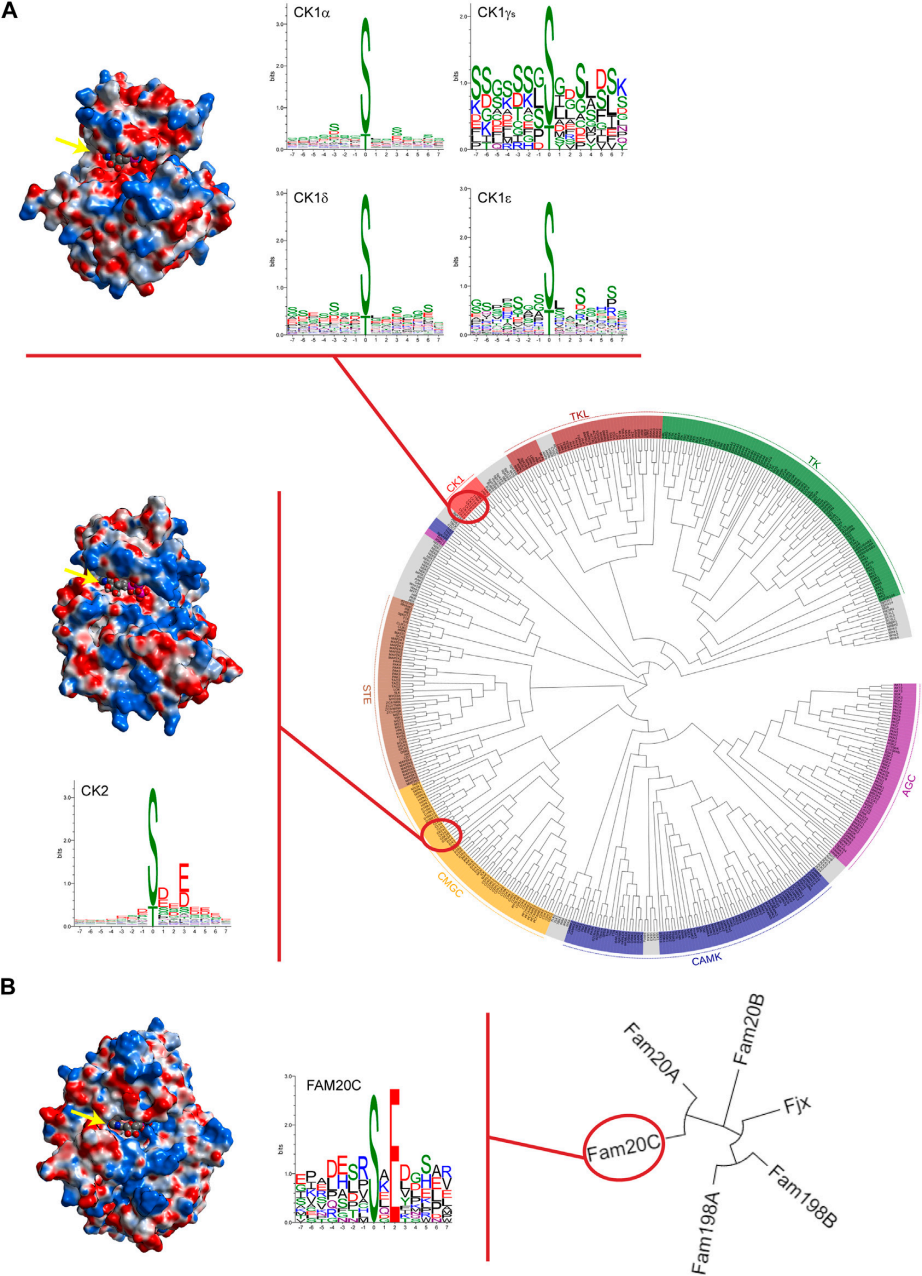
Casein kinases at a glance. **(A)** Phylogenetic dendrogram of the human kinome in which major groups of canonical protein kinases are labelled and coloured. Protein kinases CK1 and CK2 are highlighted. In the upper part, CK1δ tridimensional structure (PDB code 7P7H) is reported as an illustrative example of the CK1 family comprising six different isoforms and WebLogos for each isoform are depicted. For CK1γ isoforms (namely CK1γ1, CK1γ2, and CK1γ3), a single WebLogo of merged substrate sequences phosphorylated by the three isoforms is displayed. In the lower part, crystal structure of α catalytic subunit of protein kinase CK2 (PDB code 3WOW) and WebLogo analysis of CK2 holoenzyme’s known substrates are reported. **(B)** Phylogenetic tree of the Four-Jointed family of atypical protein kinases. Due to their altered amino acid sequences in respect to the canonical kinases, none of these atypical kinases has been included in the human kinome. On the left, Fam20C crystal structure (PDB code 4KQB) and its own WebLogo. Fam20C is the Genuine Casein Kinase (G-CK) responsible for phosphorylating the majority of secreted proteins. WebLogo analysis was performed on human protein sequences phosphorylated by casein kinases as reported on PhosphositePlus^®^ database (www.phosphosite.org). Note that WebLogos evidence the acidophilic nature of casein kinases as suggested by the residues surrounding the phosphorylation site (in position “0”), either downstream or upstream. Analytic Connolly’s electrostatic charge distribution surface was obtained through the MOE 2020.09 software. Blue colour indicates positive surface charges whereas red colour indicates negative surface charges. It is worth to note that in the case of CK2, an extensive basic region (in blue) is present both above and below the ATP binding site (indicated by yellow arrow) protruding towards the right side. Conversely, in the case of Fam20C and CK1δ, the basic region is much less extensive and is located, in both cases, in a region underlying the ATP pocket; in addition, for CK1 only, a positively charged region is present on the upper left side. The trend of the arrangement of the basic residues evidenced by the electrostatic surfaces are in perfect agreement with the consensus sequences revealed by the WebLogos. Phylogenetic trees were drawn using EvolView program (www.evolgenius.info).

In such a context, so called “casein kinases,” a heterogeneous category of phylogenetically distant acidophilic protein kinases (namely CK1, CK2, and Fam20C, see Figure 1 for an overview) that share the ability to phosphorylate casein *in vitro*, represent an appealing Research Topic in which unanticipated pathophysiological aspects of these enzymes are continuously emerging.

The extraordinary pleiotropicity of casein kinases reflects their involvement in a wide variety of both physiological and pathological processes. Indeed, the dramatic effects of casein kinases dysregulation have been observed in several pathologies, including cancer, neurodegenerative disorders, viral and parasite infections, and biomineralization diseases among others (Venerando et al., 2014). Therefore, the specific tuning of casein kinases is considered instrumental to counteract many pathological conditions.

This Research Topic, composed of thirteen original research papers, five review articles, and one mini review, provides an up-to-date snapshot of the ongoing research in the casein kinases field shedding light on their implication in human diseases as well as on innovative strategies to modulate their activity.

## Protein kinase CK1

Casein kinase 1 (CK1 or CKI) is a family of protein kinases of the human kinome (Figure 1A) that function in diverse biological pathways, and the regulation of which is just beginning to be understood.

In this Research Topic, novel information on the role of CK1 in Alzheimer’s disease, in the circadian rhythm, and in Leishmaniasis is provided, as well as a review on the role of casein kinases in neurodegenerative diseases. Finally, cutting-edge methods to generate isoform specific CKI modulators are proposed.

Alzheimer’s disease is a degenerative disease that affects the brain. One of the main pathological events in Alzheimer’s disease is the generation of neurofibrillary tangles that are formed when the tau protein becomes hyperphosphorylated and aggregates. It is suspected that members of the protein kinase CK1 family are involved in the pathogenesis of Alzheimer’s disease, as they can phosphorylate tau. Roth et al. analysed recombinant tau441 phosphorylated *in vitro* by CK1δ *via* mass spectrometry and identified ten potential phosphorylation sites, five of which are associated with Alzheimer’s disease. *In vitro* kinase assays and two-dimensional phosphopeptide analyses were performed with tau441 phospho-mutants confirming that Alzheimer’s disease-associated residues Ser68/Thr71 and Ser289 are CK1δ-specific phosphorylation sites. Finally, the use of an *in vitro* tau aggregation assay suggested that CK1δ represents a good target for the prevention of Alzheimer’s disease.

An overview of the impact of CK1, TTBK, and CK2 kinases on neurodegenerative diseases as well as a summary of their substrates and inhibitors has been presented by Baier and Szyszka.

An interesting observation is reviewed by Francisco and Virshup. The authors highlighted that, among kinases, CK1 is unique in that substrate specificity is altered by its own phosphorylation state. A telling example is provided by the PERIOD2 (PER2) phosphoswitch: in this case, CK1δ/ε kinase activity can be varied between three different substrate motifs to regulate the circadian clock.

Compelling novel results are presented by Smirlis et al., which developed a technology that allows efficient identification of substrates in the context of Leishmaniasis. Leishmaniasis is a severe public health problem caused by the protozoan Leishmania. To get insights into the functions of L-CK1.2 in the macrophage, the systematic identification of its host substrates was performed, leading to the identification of 225 host substrates as well as a potential novel phosphorylation motif for CK1. The L-CK1.2 substratome is enriched upon Leishmania infection, suggesting that L-CK1.2 might be a master regulator of this process.

Sunkari et al. offer a cutting-edge proposal to find isoform specific modulators, an especially thorny issue considering the high degree of conservation of the ATP-binding site among CK1 isoforms and the fact that all available CK1 inhibitors are ATP site-directed. The authors proposed that the DNA-encoded library (DEL) technology might represent a valuable approach to uncover allosteric modulators instead of ATP competitors.

Finally, in the research scenario of computational methodologies for the identification of novel molecules with therapeutic potential, Pavan et al. investigated CK1δ as a target for the development of an implemented version of Autogrow4 docking software with an alternative scoring function based on protein-ligand interaction fingerprints.

## Protein kinase CK2

The constitutively active casein kinase 2 (CK2 or CKII) has been among the first identified protein kinases (Venerando et al., 2017). CK2, belonging to the class of CMGC protein kinases, is part of the human kinome. The CK2 hetero-tetrameric enzyme, composed of two catalytic (α and/or α’) and two regulatory (β) subunits encoded by CSNK2A1 (and/or CSNK2A2) and CSNK2B genes, respectively, is considered a “lateral player” in several signaling pathways (Borgo et al., 2021). Notably, the “regulatory” functions of β subunits are not restricted at preserving the enzyme stability and driving the substrates specificity. Conversely, it has been proposed that unbalanced expression of CK2 subunits prompts epithelial to mesenchymal transition fostering cancer invasion and spreading. In particular, Filhol et al. showed that CK2β loss promotes focal adhesion formation and invasion by triggering pTyr signaling and activation of the FAK1-Src-PAX1 pathway suggesting a key role of CK2β in controlling cancer progression and metastasis formation.

An interesting role of copper as an enhancer of CK2 activity has recently emerged. Chojnowski et al. suggested that the ability of copper to increase CK2 activity occurs only if the phosphate donor is ATP and not GTP. This effect is counteracted by the copper chelator Cu-ATSM and increases or decreases of intracellular copper level raises or reduces CK2 signaling. Note that the two identified residues essential for copper-binding (Met153 and His154) are conserved in many kinases and consequently further systematic studies are required to assess the possible role of copper as a physiological regulator of other kinases.

Although CK2 malfunctions have been described in many different pathologies, its role in the pathogenesis of various cancers deserves special attention, as it appears to correlate to almost all malignant hallmarks. CK2 is overexpressed in many cancer cells where it potentiates other oncogenic signals and overall sustains tumorigenesis. Especially in haematological malignancies, overexpression and pro-oncogenic functions of CK2 have been widely reported. As an example, Manni et al. demonstrated that in mantle cell lymphoma, overexpressed CK2 sustains BCR signaling and Bcl-2 family proteins. Note that BCR signaling and Bcl-2 related pro-survival pathways are considered rational therapeutic targets in B cell derived lymphomas; most importantly, CK2 inhibition enhances cytotoxicity induced by the combination of chemical inhibitors directed against both pathways.

Downregulating a constitutively active enzyme generally overexpressed in malignant cells, represents a reasonable strategy to treat related diseases (Borgo et al., 2021) and during the last decades several CK2 inhibitors have been proposed. Two of them, namely CX-4945 and CIGB-300, are already in human clinical trials as anticancer drugs. In this respect, the research on the peptide-based inhibitor CIGB-300 by Rosales et al. has disclosed its antileukemic effect. Coupling LC-MS/MS data and bioinformatics in two human cell lines (HL-60 and OCI-AML3), the authors revealed that CIGB-300 promotes oxidative stress and ROS production, which might play a relevant role on CIGB-300-induced apoptosis.

From a different point of view, metal-oxide polyanionic clusters called polyoxometalates (POMs) have been previously identified as CK2 inhibitors in the nanomolar range. Nevertheless, their mechanism of action was a matter of study until today. In fact, a recent study by Fabbian et al. on ruthenium-based polyoxometalate (Ru4POM) has deciphered the mechanism of action of these unconventional inhibitors demonstrating that it involves both CK2ɑ and CK2ɑ2β2 tetramer with which the inhibitor forms stable complexes by interacting with the substrate binding site.

The employment of specific CK2 inhibitors has found an additional application for the identification and validation of CK2 substrates. Gyenis et al., using quantitative phosphoproteomics of cells expressing inhibitor-resistant CK2α mutant in comparison with its wild type counterpart, have been able to identify *bona fide* targets among CK2 putative substrates, thus overcoming the caveat that ATP site-directed inhibitors invariably display off-target effect given the high conservation of the ATP site among protein kinases. Interestingly, this strategy has the potential to be successfully applied also to the identification of *bona fide* substrates of other protein kinases.

A different perspective is given by the recent discovery that thymidylate synthase (TS) and dihydrofolate reductase (DHFR) are phosphorylated by CK2, paving the road toward an in-depth study of the role of CK2 in the di- and trimolecular complexes formation between TS, DHFR and hydroxymethyltransferase (SHMT), responsible for the thymidylate synthesis. In particular, Wińska et al. demonstrated that CK2 inhibition by CX-4945 leads to increased level of DHFR and SHMT possibly through a decrease of their ubiquitination; on the contrary, CK2 inhibition has an opposite impact on TS causing a decrease of the protein level. Moreover, the phosphorylation of TS increases the stability of its complex with SHMT1 and DHFR in comparison to non-phosphorylated TS.

Considering the high number of substrates and the ubiquity of CK2, it appears clear that CK2 regulates many cellular functions and its activity is considered essential for cellular homeostasis in different tissues. It has been suggested that CK2 (at least its α subunit) is more abundant in brain than in other tissues and its role in the brain is recognized as vital for neurological development and functioning. As an example, Nguyen et al. proposed that CK2 signaling is responsible for the activation of a spatiotemporal cascade pathway that mediates ischemic injury in white matter.

It should be stressed that several neurodegeneration-related CK2 targets have been identified so far suggesting a link (either pathogenic or protective) between CK2 and different well-acknowledged neurodegenerative diseases. Recently, two newly identified rare neurodevelopmental disorders have been associated to mutations in the genes encoding either the α catalytic (in the Okur-Chung neurodevelopmental syndrome, OCNDS) or the β regulatory (in the Poirier-Bienvenu neurodevelopmental syndrome, POBINDS) subunits of CK2. In OCNDS patients, one of the most frequently observed mutation is lysine-198 to arginine exchange (K198R) in the P+1 activation loop of CK2α. Caefer et al. demonstrated that K198R mutant is still an active enzyme even though it presents a different substrate specificity in comparison to the wild type kinase. The change in the kinase specificity is mainly due to a shift of the anion binding site harboured by the P+1 loop rather than by an altered interaction between the catalytic with the non-catalytic subunits in the CK2 holoenzyme, as revealed by the K198R mutant crystal structure presented by Werner et al.

Although the two syndromes display very similar clinical manifestations, it has been possible to rationalize the diagnosis based on clinical symptomatology in comparison with preclinical and biochemical CK2 characterization, as described by Ballardin et al. Finally, Unni et al., integrating information on the CK2 genes mutations discovered in OCNDS and POBIND patients with experimental data, have provided useful predictive tools that allow a better understanding of the functional and structural consequences of such mutations.

## Atypical protein kinase Fam20C

Family with sequence similarity 20 member C also known as Fam20C or DMP4 is a protein, recently identified as the Genuine Casein Kinase (G-CK or GEF-CK), especially abundant in the Golgi apparatus of the lactating mammary gland (Tagliabracci et al., 2012). Fam20C is an atypical serine protein kinase that possess little sequence similarity to canonical protein kinases present in the human kinome. Fam20C phosphorylates not only casein but also highly acidic secreted proteins with the common consensus motif S-x-E/pS (Lasa-Benito et al., 1996). Although Fam20C is far from the other two casein kinases both structurally and phylogenetically (Figure 1), it shares with them the extraordinary pleiotropicity. Indeed, Fam20C is the major contributor in the generation of the human phosphosecretome, the pool of phosphorylated secreted proteins (Tagliabracci et al., 2015). From this point of view, Xu et al. reviewed the structure and biological functions of Fam20C with special reference to its ability to phosphorylate secreted proteins. The important role of Fam20C in biomineralization has been addressed being Fam20C implicated in the phosphorylation of the small integrin binding ligand-N-linked glycoproteins (SIBLINGs), dentin matrix protein 1, matrix extracellular phosphoglycoprotein, osteopontin, bone sialoprotein, and dentin sialophosphoprotein. The best-known and dramatic consequence of Fam20C aberrant function in biomineralization is epitomized by the Raine syndrome, a lethal osteosclerotic bone dysplasia. However, the phosphorylation of multiple substrates by Fam20C justifies its role in many other life processes and its implication in several diseases, such as in smooth muscle cell calcification and frontotemporal dementia, have been discovered recently. In addition, cardiovascular implications of Fam20C is inferred from the phosphorylation of calsequestrin 2, matrix interacting molecule 1, and fibroblast growth factor 23, connecting this atypical protein kinase to heart and neurovascular diseases. Finally, the phosphorylation by Fam20C of insulin-like growth factor binding proteins and osteopontin reveals the role of Fam20C in tumorigenesis. Notwithstanding, the regulation mechanism of Fam20C activity is still a matter of study and, unfortunately, no contributions have been submitted to this Research Topic to shed additional light on this intricate matter. In the past, two possible mechanisms not mutually exclusive have been proposed. One is the association of Fam20C with Fam20A, a Fam20C paralog with no kinase activity by itself but able to increase the activity of Fam20C (Cui et al., 2015); another mechanism appears to exploit sphingosine and some related compounds to stimulate Fam20C kinase activity (Cozza et al., 2017). In this respect, it should be noted that stimulation rather than inhibition of this atypical protein kinase appears to represent a valuable pharmacological strategy. Indeed, Fam20C-linked diseases are generally a consequence of its defective expression or activity. We hope that this review stimulates the scientific community to deepen the research on Fam20C as an important therapeutic target.
